# Supplementary material for: A Negative Regulatory Role for RKIP in Breast Cancer Immune Response
Source: Cancers (Basel). 2022 Jul 24;14(15):3605. doi: 10.3390/cancers14153605 (PMC9330697; doi:10.3390/cancers14153605)
Supplement: Supplementary file 1 [file cancers-14-03605-s001.zip › cancers-1820402-supplementary.pdf]

Supplementary materials. The purpose of the analysis in supplementary materials was to determine is the patterns found for all of the breast cancer samples were maintained in the different breast cancer subtypes.

Methods. The brca_tcga_pan_can_atlas_2018 files were downloaded from https://www.cbioportal.org/. The expression data were extracted from the file data_mrna_seq_v2_rsem_zscores_ref_all_samples. After importing the data into the R platform, rows with no gene names or with missing values were removed. Correlations between PEBP1/RKIP mRNA expression and all other genes were calculated using the cor.test function. The PAM50 cancer subtypes were extracted from the file data_clinical_patient. The process was repeated by calculating the correlations for each subtype. Numbers of samples/subtype: LumA, 499; LumB, 197; Basal, 171; Her2, 76; Normal, 36; Unclassified, 103.

The correlation tables were converted to .rnk files for submission to GSEA. The gene sets were the 50 Hallmark gene sets contained in h.all.v.7.5.1 symbols. The results were similar to the results reported in the manuscript, with many immune-related categories.

Results.

All	LumA	LumB	Her2	Basal	Normal
INFLAMMATORY_RESPONSE	EPITHELIAL_MESENCHYMAL_TRANSITION	EPITHELIAL_MESENCHYMAL_TRANSITION	MITOTIC_SPINDLE	INTERFERON_GAMMA_RESPONSE	EPITHELIAL_MESENCHYMAL_TRAN
EPITHELIAL_MESENCHYMAL_TRANSITION	INFLAMMATORY_RESPONSE	UV_RESPONSE_DN	UV_RESPONSE_DN	INFLAMMATORY_RESPONSE	UV_RESPONSE_DN
INTERFERON_GAMMA_RESPONSE	UV_RESPONSE_DN	INFLAMMATORY_RESPONSE	G2M_CHECKPOINT	EPITHELIAL_MESENCHYMAL_TRAN	INFLAMMATORY_RESPONSE
TNFA_SIGNALING_VIA_NFKB	KRAS_SIGNALING_UP	KRAS_SIGNALING_UP	TNFA_SIGNALING_VIA_NFKB	TNFA_SIGNALING_VIA_NFKB	ALLOGRAFT_REJECTION
ALLOGRAFT_REJECTION	MITOTIC_SPINDLE	MITOTIC_SPINDLE	PROTEIN_SECRETION	ALLOGRAFT_REJECTION	KRAS_SIGNALING_UP
IL6_JAK_STAT3_SIGNALING	TNFA_SIGNALING_VIA_NFKB	INTERFERON_GAMMA_RESPONSE	TGF_BETA_SIGNALING	INTERFERON_ALPHA_RESPONSE	COMPLEMENT
UV_RESPONSE_DN	ALLOGRAFT_REJECTION	TGF_BETA_SIGNALING	ANDROGEN_RESPONSE	COMPLEMENT	IL6_JAK_STAT3_SIGNALING
KRAS_SIGNALING_UP	INTERFERON_GAMMA_RESPONSE	TNFA_SIGNALING_VIA_NFKB	APICAL_SURFACE	IL6_JAK_STAT3_SIGNALING	MITOTIC_SPINDLE
MITOTIC_SPINDLE	IL6_JAK_STAT3_SIGNALING	ALLOGRAFT_REJECTION	INFLAMMATORY_RESPONSE	UV_RESPONSE_DN	TNFA_SIGNALING_VIA_NFKB
COMPLEMENT	TGF_BETA_SIGNALING	IL6_JAK_STAT3_SIGNALING	IL6_JAK_STAT3_SIGNALING	KRAS_SIGNALING_UP	INTERFERON_GAMMA_RESPONSE
INTERFERON_ALPHA_RESPONSE	COMPLEMENT	HEDGEHOG_SIGNALING	E2F_TARGETS	IL2_STAT5_SIGNALING	TGF_BETA_SIGNALING
TGF_BETA_SIGNALING	IL2_STAT5_SIGNALING	COMPLEMENT	ESTROGEN_RESPONSE_EARLY	ANDROGEN_RESPONSE	IL2_STAT5_SIGNALING
IL2_STAT5_SIGNALING	APICAL_JUNCTION	PROTEIN_SECRETION	HEDGEHOG_SIGNALING	COAGULATION	PROTEIN_SECRETION
G2M_CHECKPOINT	HEDGEHOG_SIGNALING	ANDROGEN_RESPONSE	HYPOXIA	APOPTOSIS	ANGIOGENESIS
APICAL_JUNCTION	INTERFERON_ALPHA_RESPONSE	IL2_STAT5_SIGNALING		TGF_BETA_SIGNALING	HEDGEHOG_SIGNALING
HEDGEHOG_SIGNALING	ANGIOGENESIS	G2M_CHECKPOINT		ANGIOGENESIS	ANDROGEN_RESPONSE
ANGIOGENESIS	ANDROGEN_RESPONSE	INTERFERON_ALPHA_RESPONSE		PROTEIN_SECRETION	APOPTOSIS
PROTEIN_SECRETION	G2M_CHECKPOINT	HYPOXIA		APICAL_JUNCTION	APICAL_JUNCTION
ANDROGEN_RESPONSE	APOPTOSIS	APICAL_JUNCTION		MITOTIC_SPINDLE	COAGULATION
HYPOXIA	COAGULATION	ANGIOGENESIS		HYPOXIA	INTERFERON_ALPHA_RESPONSE
APOPTOSIS	NOTCH_SIGNALING	NOTCH_SIGNALING		APICAL_SURFACE	PI3K_AKT_MTOR_SIGNALING
COAGULATION	WNT_BETA_CATENIN_SIGNALING	WNT_BETA_CATENIN_SIGNALING		ESTROGEN_RESPONSE_EARLY	HEME_METABOLISM
NOTCH_SIGNALING	PROTEIN_SECRETION			HEME_METABOLISM	
APICAL_SURFACE	HYPOXIA				
	APICAL_SURFACE				

Supplementary Table S1. GSEA results for all samples and subtypes.

The GSEA results classifies gene sets with positive or negative correlation with PEBP1/RKIP expression. We are interested in sets that are negatively related to PEPB1 expression. About half of the 50 sets are positively related and about half are negatively related for all the subtypes. The gene sets with FDR q-values < 0.05 are shown in the table. This number was between 22 and 25 for all the subtypes except for Her2, which had only 14 gene sets below this cutoff.

The sets are ranked, smallest q-values at top (q-values not shown). The sets that are related to immune response are highlighted in yellow. Conclusion: the negative association of PEBP1/RKIP expression with immune function genes holds for all breast cancer subtypes.

Venn diagrams. These lists in Supplementary Table S1 were used to create Venn diagrams to visually display the overlaps between categories. The R program Vennerable was used to create the diagrams.

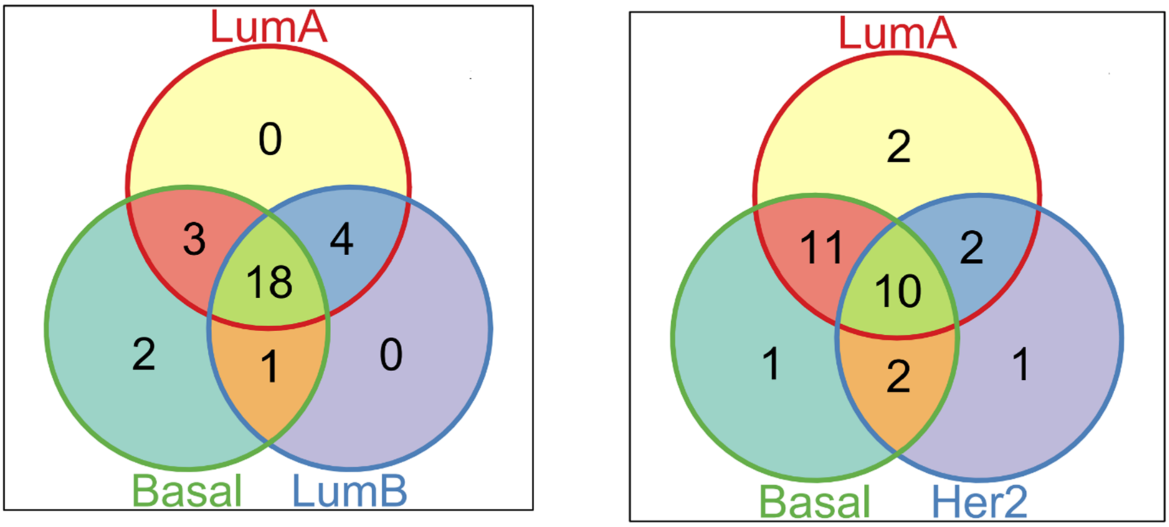

Supplementary Figure S1. A. Venn diagram showing overlap between the LumA, LumB, and Basal subtypes. B. Venn diagram showing overlap between the LumA, Her2, and Basal subtypes
